# Supplementary material for: The interactome of intact mitochondria by cross-linking mass spectrometry provides evidence for coexisting respiratory supercomplexes
Source: Mol Cell Proteomics. 2017 Dec 8;17(2):216–32. doi: 10.1074/mcp.RA117.000470 (PMC5795388; doi:10.1074/mcp.RA117.000470)
Supplement: Supplemental Data [file supp_RA117.000470_133922_0_supp_23978_fzffwf.zip › spectra_annotation/mito_spectra_annotation/438-1-8-1-6-1.pdf]

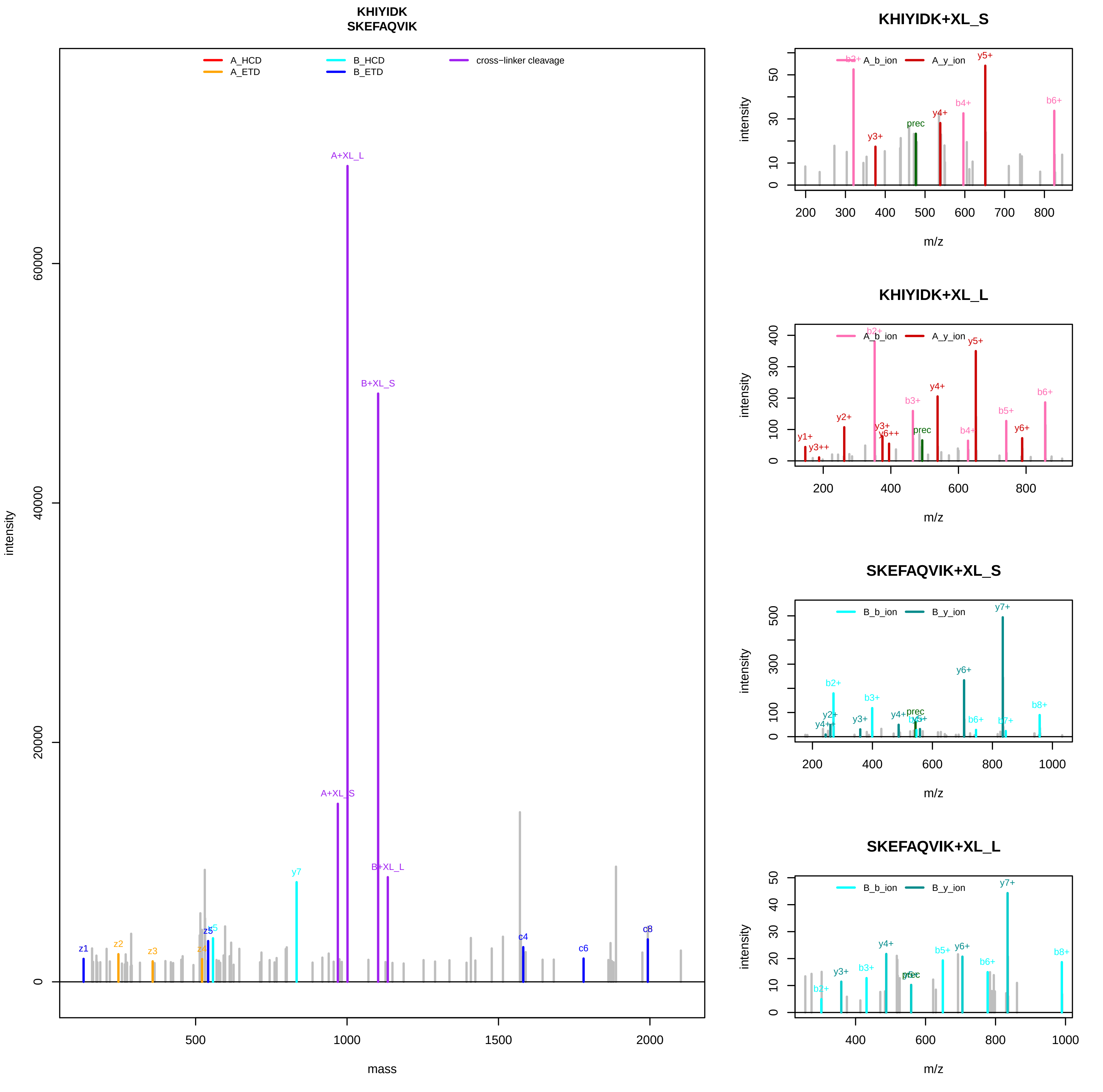
KHIYIDK
SKEFAQVIK
A_HCD
A_ETD
B_HCD
B_ETD
cross−linker cleavage
A+XL_L
B+XL_S
A+XL_S
B+XL_L
y7
z1
z2
z3
z4
z5
y5
c4
c6
c8
intensity
mass
0
20000
40000
60000
500
1000
1500
2000
KHIYIDK+XL_S
A_b_ion
A_y_ion
b2+
y5+
b4+
b6+
y4+
prec
y3+
intensity
m/z
KHIYIDK+XL_L
b2+
y5+
y4+
b3+
b6+
b5+
y2+
y3+
y6++
prec
b4+
y6+
y1+
y3++
SKEFAQVIK+XL_S
B_b_ion
B_y_ion
y7+
y6+
b2+
b3+
b8+
y2+
y4++
y3+
y4+
prec
b5+
b6+
b7+
SKEFAQVIK+XL_L
y7+
y4+
y6+
b5+
b8+
b3+
y3+
b6+
prec
b2+
